# Chronic Inflammatory or Chronic Inflammatory Demyelinating Polyradiculoneuropathy?

**DOI:** 10.3389/fneur.2022.862335

**Published:** 2022-04-04

**Authors:** Jean-Michel Vallat, Nathalie Deschamps, Philippe Corcia, Laurent Magy, Stéphane Mathis

**Affiliations:** ^1^Department and Laboratory of Neurology, National Reference Center for “Rare Peripheral Neuropathies”, University Hospital of Limoges (CHU Limoges), Dupuytren Hospital, Limoges, France; ^2^Department of Neurology, ALS Center, University Hospital of Tours (CHU Tours–Bretonneau Hospital), Tours, France; ^3^Department of Neurology (Nerve-Muscle Unit), AOC National Reference Center for Neuromuscular Disorders, ALS Center, University Hospital of Bordeaux (CHU Bordeaux), Pellegrin Hospital, Bordeaux, France

**Keywords:** polyradiculoneuropathy, node of Ranvier, axonal, demyelinating, inflammation, nodopathy

## Introduction

The second revision of the “Chronic Inflammatory Demyelinating Polyneuropathy (CIDP) diagnostic criteria,” now called “*European Academy of Neurology (EAN) /Peripheral Nerve Society (PNS) Guideline on diagnosis and treatment of chronic inflammatory demyelinating polyradiculoneuropathy*,” has been published recently (1). These new criteria (EAN/PNS 2021) need to have at least as good diagnostic accuracy as the former ones (EFNS/PNS 2010) (2). This update mostly relies on electrodiagnostic results for detection of peripheral nerve demyelination. This pathological feature also induces or is accompanied by axonal involvement of variable severity which needs to be carefully analyzed and discussed in the diagnosis of “CIDP.”

## Segmental Demyelination Characterizes CIDP Nerve Lesions, But…

In a seminal paper (1975), Dyck et al. isolated (from chronic idiopathic polyneuropathies) a subgroup of 53 patients who could be identified clinically as having a grossly symmetric, sensorimotor polyneuropathy, which they called “*Chronic Inflammatory Polyradiculoneuropathy*” (CIP) (3). In this paper, the authors discussed several other terms that might best define this syndrome characterized by its natural history, clinical signs, electrophysiological anomalies, CSF changes and pathological lesions. At that time, among different other possibilities, Dyck et al. estimated that the word “*demyelinating*” was too “*inclusive*.” In fact, the authors indicated that the first evidence of damage was segmental demyelination, but the most frequent abnormality of the nerve biopsies (NB) of 26 patients was degeneration of myelinated fibers into linear rows of myelin ovoids and balls (that are characteristic features of ongoing axonal damage).

Before 1975, many patients were reported as having “*recurring and relapsing neuritis*” (chronic progressive peripheral neuropathies were rarely reported). In 1982, Dyck et al. used, for the first time, the term “CIDP” (4), by analogy with “Acute Inflammatory Demyelinating Polyradiculoneuropathy” (AIDP), and demonstrated its sensitivity to steroids. CIDP (CIP) progressively develops over more than 8 weeks, distinguishing this condition from “Guillain-Barré Syndrome” (GBS) which has an acute onset (<4 weeks) (6, 7); both conditions (GBS and CIDP) are thought to have an auto-immune basis. Electrophysiologically, criteria of segmental demyelination (such as slowing of nerve conduction velocities, prolongations of distal latencies, conduction blocks and temporal dispersion mainly of motor fibers) are identified, although they are not invariable findings in CIP (1). The pathological features mainly involve myelinated fibers and are classically characterized by segmental demyelination (that is now sometimes called “internodopathy”), corresponding to randomly distributed foci of acquired demyelination between two nodes of Ranvier, or internode. As noted above, the presence of axonal lesions is also usually significant [about 25% of the nerve fibers in the first paper by Dyck et al. (3)].

## Axonal Involvement Does not Exclude CIDP

We think that the word “*demyelinating*” (referring to the “D” of “CIDP”) might be too restrictive; many neurologists and electrophysiologists noting the presence of significant axonal lesions may miss the diagnosis of CIP which, in most cases, respond to immunosuppressive/immunomodulatory treatments: positive randomized control trials have confirmed the efficiency of intravenous immunoglobulin (IVIg) and plasma exchanges in this disorder (5, 6).

There is no gold standard test to definitively diagnose CIDP. Nevertheless, the second revision of CIDP criteria (EAN/PNS 2021) should be very helpful (1). In routine practice, the main objective of electrodiagnostic study is to demonstrate a demyelinating profile (which may be very difficult to obtain when axonal involvement becomes prominent). The above-mentioned classical abnormalities in favor of demyelination are sometimes not observed because of the limitations of the electrophysiological tests, giving rise to a mixed axonal and demyelinating profile, especially in patients with a long-standing disease. Although these patients do not meet electrodiagnostic criteria for demyelination, it is of great interest that the EAN/PNS diagnostic criteria now allow confirming the diagnosis of CIP, using clinical characteristics and response to treatment. Recently, Oh et al. presented diagnostic criteria, which they proposed to be characteristic of another type of auto-immune peripheral neuropathy, which they call “*Chronic Inflammatory Axonal Polyneuropathy*” (CIAP) (7); nevertheless, this entity has to be confirmed by independent studies.

## The Various Mechanisms of Axonal Degeneration and Loss

The causes of axonal loss, which correlate with permanent clinical disability are poorly understood; nevertheless, several mechanisms are plausible. Axonal loss is usually considered as a secondary event in demyelinating disorders and its severity has implications for the diagnosis and prognosis; nevertheless recent studies indicate that axonal damage might also occur early in the disease (8). CIDP involves mainly myelinated fibers, but unmyelinated nerve fibers are also involved, probably due to a heterogeneous immunological pathogenesis and the severity of the inflammatory process (9).

It is known that the lesions of auto-immune peripheral neuropathy (characterized by inflammatory cells and edema) are most severe at the nerve roots and the proximal parts of the nerves, and may also be diffuse throughout the length of peripheral nerves; Dyck et al. have suggested that these lesions might determine transection of nerve fibers and induce distal Wallerian degeneration (3). Such chronic auto-immune peripheral neuropathies are classically considered as “primary demyelinating.” In fact, after a variable and unpredictable course, any chronic demyelinating peripheral neuropathy (whatever the cause) induces a secondary axonal loss of varying intensity. It is why CIDP should be envisaged during investigation of any chronic multifocal or generalized peripheral neuropathy of unknown cause. In such cases, as mentioned in the recent EAN/PNS criteria (1), MRI and ultrasound examinations of nerve roots and trunks are now included as supportive elements; nevertheless these tools do not seem capable of providing definitive information about axonal lesions and prognosis. In the recently updated criteria, it is indicated that, in cases where CIDP cannot be confirmed with the clinical, laboratory, imaging, and electrodiagnostic examinations (or in cases where CIDP is suspected, but there is little or no response to treatment), nerve biopsy (NB) can be of value (1). It concerns a sensory nerve which can be the sural nerve or the superficial peroneal nerve; sometimes the superficial radial nerve may be taken when symptoms predominate in the upper limbs. By this technique, myelin and axonal involvement can be specifically examined: lesions in favor of the diagnosis of CIDP have to be considered as probable, but not absolutely specific. Pathologic hallmarks include demyelination and remyelination, which sometimes may be discrete and have to be demonstrated even in the presence of severe axonal loss. Segmental demyelination is better observed on teased nerve fiber preparations and by electron microscopic examination (EME). EME can detect macrophages penetrating a Schwann cell cytoplasm and dissociating myelin lamellae, which support a dysimmune process, as initially described by Prineas (10). Macrophages may not only destroy myelin, but also axons that appear shrunken.

## When the Axonopathy is Induced by a Nodo-Paranodopathy

Some patients diagnosed (according to electrophysiological criteria) as “CIDP” were found to have antibodies (mainly IgG4, unable to activate complement) against some components of the paranodal junctions: Neurofascin-155 (NF155), Contactin-1 (CNTN1), and Contactin-associated protein-1 (Caspr1). These patients usually present a peripheral neuropathy with sub-acute onset, chronic severe clinical course (with axonal degeneration and loss on NB) and unresponsiveness to IVIg therapy (11). The lesional mechanisms, in these cases, do not involve macrophage-associated damage. Furthermore, EME of the normal paranode shows that myelin sheath is tightly attached to the axon by specific junctions, the transverse bands (TB), comprising certain proteins: CNTN1 and Caspr1 that are located on the axon, NF155 on the myelin sheath. In patients with a so-called “paranodopathy” (who have circulating antibodies against some paranodal proteins), high magnification study of the paranodal regions shows an absence of TB at paranodes. So, in these areas, there is a loss of attachment of the myelin loops to the axon, and an irregular widening of the periaxonal space (by comparison to controls). The axon-myelin sheath attachment is destroyed, inducing a terminal loop detachment, thus initiating the lesional process characterized by retraction, without evidence of true demyelination-remyelination, but resulting in widening of the node with consequent increased nodal capacitance and dilution of the capacitive current over a larger surface. This probably explains why these patients, although not having a true demyelinating neuropathy most often have electrodiagnostic data suggestive of primary demyelination. However, axonal injury has been described in the NB of these patients, explaining the reduced amplitude of distal CMAP and spontaneous muscle activity at needle examination. So, some patients are now considered as presenting a “nodoparanodopathy” which finally could correspond to a CIP subtype. It has been suggested that such cases should no longer be classified as CIDP (11), which may be an additional reason for using the terminology “CIP.” Otherwise, we have identified in nerve samples from a few patients both types of lesions: “internodopathies” and “paranodopathies.” Although the proportion of patients with IgG4 and IgG3 against paranodal junction and node of Ranvier components appears small (probably <5% of the CIDP patients), their detection may be decisive for diagnosis and treatment with drugs such as rituximab. A similar mechanism has been advanced in patients presenting axonal sensorimotor and pure sensory peripheral neuropathy associated with anti-sulfatide antibodies (12); some of these patients had NB which showed variable degrees of axonal loss, but no EME of the longitudinal sections of the nodo-paranodal areas was performed. Otherwise, ultrastructural, immunohistological, and biochemical analysis revealed widespread axonal degeneration and disruption of the axo-glial junction at the nodes of Ranvier in mice deficient for combination of galactocerebroside and sulfatide, sulfatide alone or complex gangliosides (13). These glycolipids have fundamental functions in clustering proteins on opposing membranes that are essential to maintain axo-glial integrity, as well as its normal axonal function and structure. The same remarks concerning axonal involvement apply to other auto-immune peripheral neuropathy such as “GBS,” “subacute and relapsing peripheral neuropathy subtypes,” “multifocal motor neuropathy,” “Miller-Fisher syndrome,” and “CANOMAD.” In GBS, axonal lesions are well described and may be dominant in some subtypes, such as acute motor (AMAN) or motor and sensory (AMSAN) neuropathies (11), where the internodal penetration of macrophages contributes to the axonal degeneration in the internode (14).

## Conclusions

**Figure 1:**
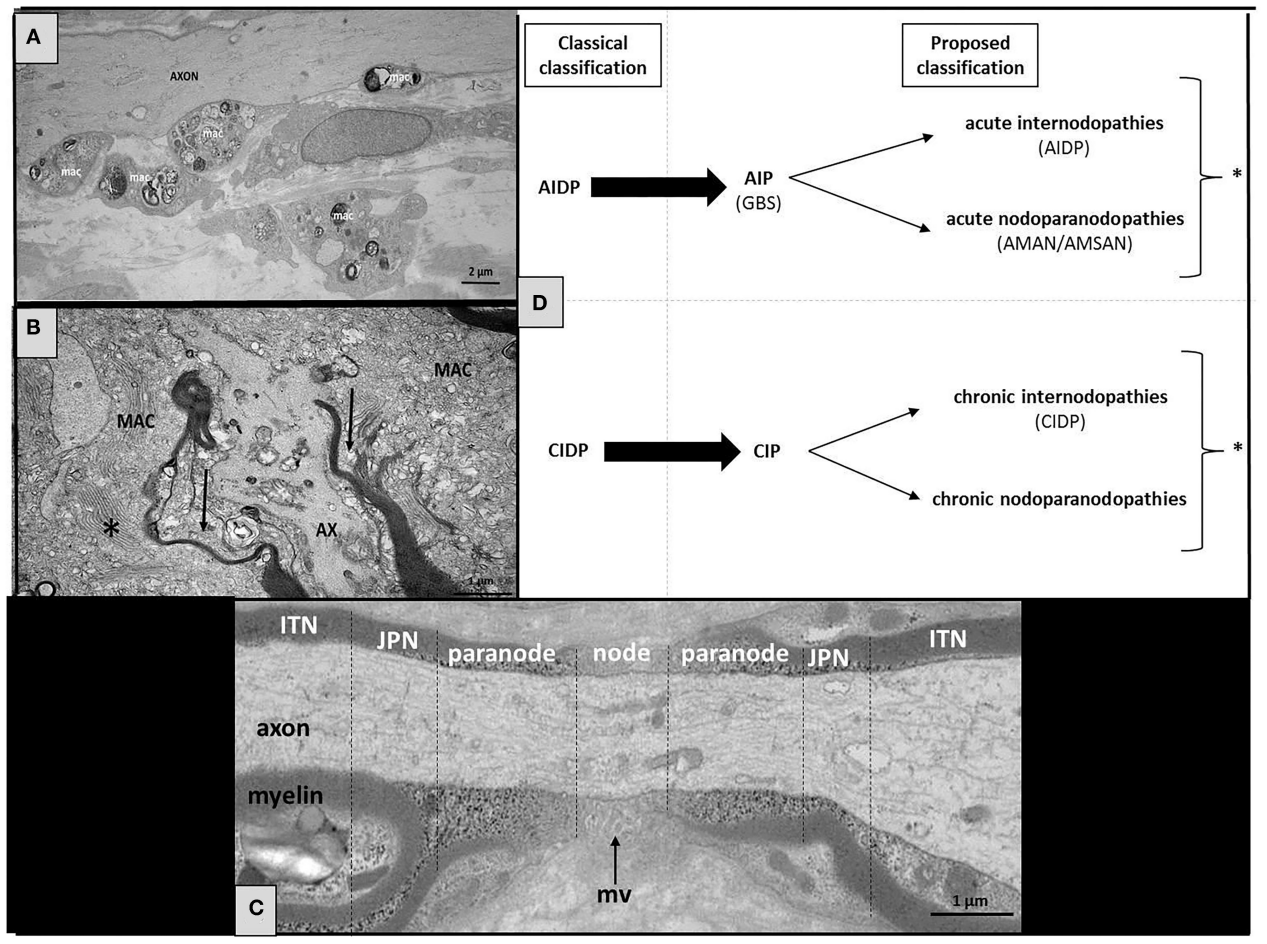
Structure of the normal and pathological node of Ranvier, with a proposed classification of the nodoparanodopathies. **(A)** Electron microscopy micrograph of longitudinal section of the sural nerve biopsy from a CIDP patient. We observe the presence of large diameter axons (AXON) completely devoid of myelin at the level of an internode. Several macrophages overloaded with myelin debris (mac) are seen in close contact to axons. **(B)** Electron microscopy micrograph of longitudinal sections of the sural nerve biopsy from a patient with nodo-paranodopathy. We observe macrophages containing vesicular-like myelin debris (*) are dissociating the paranodes (arrows). **(C)** Longitudinal section of a normal human peripheral nerve at level of the node of Ranvier area (JPN, juxta-paranode; ITN, internode; mv, microvilli). **(D)** In our proposed classification, we indicate the probabilities of where the target antigens/sites of pathology are likely to be (* some patients may present both types of lesions: internodopathy and paranodopathy).

Axonal involvement appears during the course of many chronic auto-immune peripheral neuropathy considered as “primarily demyelinating” (particularly during the course of CIDP), and also characterizes the acquired “paranodopathies.” Over time, axonal involvement becomes more severe and persistent, so that demyelinating lesions may be difficult to identify in some cases (either electrophysiologically or pathologically): such patients, sometimes wrongly considered as presenting “axonal peripheral neuropathy,” are not being treated correctly (or not treated at all). Anyway, as we stressed it recently, it is important to acknowledge that the current EAN/PNS recently published criteria have made an important step in recognizing that clinical and laboratory evidence, and ultimately response to treatment must be taken into account besides electrodiagnostic criteria, to confirm the diagnosis of CIDP (15). It must also be emphasized that pushing too far the diagnostic criteria toward axonal involvement would result in a considerable loss of specificity. Nevertheless, we believe that there are limitations in the current classification (Figure 1) and that the presence of severe axonal involvement must not preclude the diagnosis of an auto-immune peripheral neuropathy. These observations support the notion that CIDP (like GBS) represents a syndrome rather than a well-defined and homogeneous disease. Some authors recently proposed to enlarge the spectrum of CIDP by using the term “CIDP syndrome” (CIDPS), but we think it would be ultimately more appropriate to reuse the original term “CIP.” Such auto-immune peripheral neuropathies are nowadays divided into several subtypes depending on the clinical phenotype, pathophysiology, and neurophysiological features (Figure 1). This heterogeneity might be explained by the variety of antigens (and their antibodies that could serve as biomarkers) which remain to be discovered. Although there is a risk of splitting the broad spectrum of CIDP (CIP) into multiple separate entities, discovering new target antigens is of paramount importance, not only to understand their pathogenic mechanisms, but also to help in the diagnosis and treatment of these patients.

## Author Contributions

J-MV contributed with the original conception of the paper. J-MV and SM drafted the manuscript. ND, PC, and LM provided a critical review of the text. All authors contributed to the article and approved the submitted version.

## Conflict of Interest

The authors declare that the research was conducted in the absence of any commercial or financial relationships that could be construed as a potential conflict of interest.

## Publisher's Note

All claims expressed in this article are solely those of the authors and do not necessarily represent those of their affiliated organizations, or those of the publisher, the editors and the reviewers. Any product that may be evaluated in this article, or claim that may be made by its manufacturer, is not guaranteed or endorsed by the publisher.
